# Reduction of Cogging Torque in Dual Rotor Permanent Magnet Generator for Direct Coupled Wind Energy Systems

**DOI:** 10.1155/2014/987062

**Published:** 2014-08-14

**Authors:** Sivachandran Paulsamy

**Affiliations:** Department of Electrical and Electronics Engineering, Sree Sastha Institute of Engineering and Technology, Chennai 600123, India

## Abstract

In wind energy systems employing permanent magnet generator, there is an imperative need to reduce the cogging torque for smooth and reliable cut in operation. In a permanent magnet generator, cogging torque is produced due to interaction of the rotor magnets with slots and teeth of the stator. This paper is a result of an ongoing research work that deals with various methods to reduce cogging torque in dual rotor radial flux permanent magnet generator (DRFPMG) for direct coupled stand alone wind energy systems (SAWES). Three methods were applied to reduce the cogging torque in DRFPMG. The methods were changing slot opening width, changing magnet pole arc width and shifting of slot openings. A combination of these three methods was applied to reduce the cogging torque to a level suitable for direct coupled SAWES. Both determination and reduction of cogging torque were carried out by finite element analysis (FEA) using MagNet Software. The cogging torque of DRFPMG has been reduced without major change in induced emf. A prototype of 1 kW, 120 rpm DRFPMG was fabricated and tested to validate the simulation results. The test results have good agreement with the simulation predictions.

## 1. Introduction

Wind energy is clean, abundant, and renewable. Wind energy systems are widely used to generate electricity and help to improve the quality of life. The worldwide wind power installed capacity at the end of 2012 is 282.5 GW. The percentage shares of the leading countries such as China, USA, and Germany are 26.7, 21.2, and 11.1, respectively [1]. Small SAWES play a vital role in meeting the demand for electricity. The global installed capacity of small SAWES at the end of 2012 is 728.3 MW [2]. Such systems provide power supply to remote locations that are not connected to common grid. They have tremendous potential to reduce transmission and distribution losses by restricting grid expansion [3].

The permanent magnet (PM) machines provide high power density and high torque density. The design of PM machines is an iterative process with different parameter values [4]. The practical design of a PM machine includes commercial material specifications and magnetic and electric circuit analysis [5]. Permanent magnet generators became popular because they eliminate field windings and enable direct coupling of wind turbines [6]. A number of different topologies of PM wind generator were designed and compared [7]. A low speed direct coupled PM generator is an important feature that makes a SAWES robust and reliable. The FEA of a PM machine is performed in order to verify the effectiveness of the design process and validate the designed parameters. The FEA is used to determine the quantities such as flux linkage, flux density, and induced emf [8].

In PM machines, cogging torque produces both vibration and noise. Li and Slemon reduced the cogging torque to an acceptable level by selection of suitable PM machine dimensions [9]. The various methods such as the effect of machine symmetry [10], the effect of slot and pole number combination, the use of supplementary teeth and slots, optimizing magnet pole arc, and skewing and changing the width of stator slot openings [11–13] were applied to reduce the cogging torque. In addition, the cogging torque was reduced by magnet shifting method [14, 15].

In surface mounted PM machines, effect of magnet segmentation, skewing, design with different PM pole arc widths, and introduction of dummy slots in the stator teeth were practiced [16–18]. Muljadi and Green [19] investigated that, in PM generators for small wind turbines with cogging torque of more than 1 Nm, the wind turbine can never come out of stall mode and may never start. Lukaniszyn and Mlot [20] recommend that, in small PM machines, the cogging torque of less than 1 Nm is good for the starting performance of the machine.

In this paper, the cogging torque reduction was carried out in a three-phase, 1 kW, 120 rpm DRFPMG for direct coupled SAWES. Initially, three methods were applied one by one to reduce the cogging torque. The first method applied was changing the slot opening width. In this first method, the cogging torque value observed was 0.889 Nm. The second method applied was changing the permanent magnet pole arc width. In this second method, the cogging torque value observed was 1.2 Nm. The third method applied was shifting of slot openings. In this third method, the cogging torque value observed was 0.612 Nm. The cogging torque value observed from shifting of slot openings method is suitable for small wind turbine applications. However, a fourth method which is a combination of all the above three methods was applied to reduce the cogging torque further. The percentage reduction of induced emf with respect to each method of cogging torque reduction is tabulated. The FEA concerned with determination of cogging torque, reduction of cogging torque, and determination of induced emf was performed using MagNet Software. The method by which cogging torque was reduced to a level suitable for direct coupled SAWES has been implemented during prototype fabrication. The test results are presented.

## 2. Structure and Operation of DRFPMG

**Figure 1:**
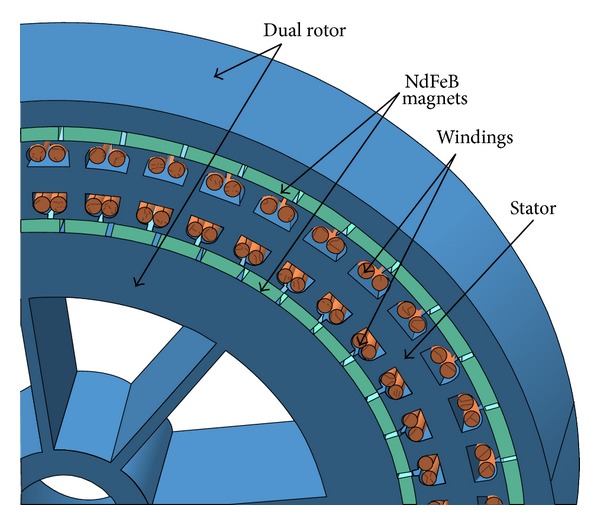
Three-dimensional view of DRFPMG section.

In the DRFPMG, an inner rotor and an outer rotor were attached together in order to rotate them at the same speed. The three-dimensional view of DRFPMG is shown in Figure 1. The high energy Neodymium Iron Boron (NdFeB) magnets were surface mounted on the inner periphery of the outer rotor and on the outer periphery of the inner rotor. The shaft of this dual rotor should be directly coupled to the SAWES. The stator was embedded between the two rotors and dual air gap was formed. The slots were present on both outer and inner periphery. The single layer three-phase windings were housed inside these slots.

**Figure 2:**
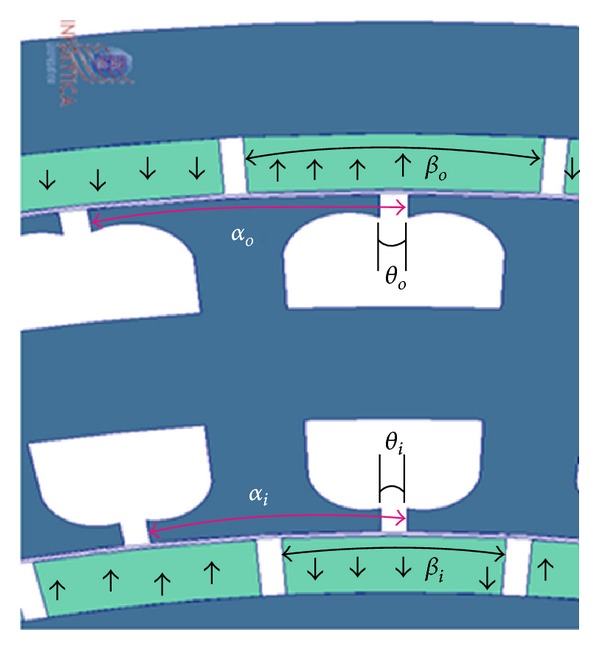
Section of DRFPMG FEA model.

The direction of magnetization of PMs is shown in Figure 2 by arrows on the magnets. The flux starting from one pole of a rotor travels along the circumference of the stator slots, links the single layer three-phase windings present in the slots, and reaches the adjacent opposite pole of the same rotor. As the dual rotor rotates the emf is induced in the windings present in the inner and outer slots of the stator simultaneously. Since both three-phase windings were connected in series, the emf produced gets added up and available at the terminals of the machine. The common back iron of the stator served as return path for the flux lines. Thus, DRFPMG worked as two conventional radial flux permanent magnet generators connected in series.

The important advantages of DRFPMG are as follows. (1) The doubled air gap associated with dual rotors can produce more power in a slightly enlarged volume. The material cost is therefore sharply reduced. (2) The DRFPMG structure is suitable for both short and long machines as it has nonslotted rotor core. (3) Both of the working surfaces of the stator core are used. This allows the DRFPMG to exploit a higher percentage of the stator winding, for the production of the output power in comparison with conventional machines. This leads to high efficiency, high ratio of diameter to length, and very short end winding. (4) The large armature reaction and low overload capability caused by slotting in the stator are compensated using surface mounted permanent magnets. (5) Nonslotted toroidal winding is possible in the stator.

## 3. Determination of Cogging Torque in DRFPMG

The cogging torque in a permanent magnet machine is given by

(1) $$\begin{array}{c} T_{\text{cog}}=-\frac{1}{2}\Phi_{g}^{2}\frac{dR}{d\theta }, \end{array}$$

where Φ_*g*_ is the air gap flux, *R* is the air gap reluctance, and *θ* is the angular position of the rotor [4].

The resultant cogging torque of inner and outer air gap of the DRFPMG depends upon the factors such as slot opening width, the PM pole arc width, and the position of slot openings. The slot opening width is inversely proportional to Carter's coefficient of the air gap and directly proportional to the magnetic flux density in the air gap. The change in slot opening width creates a phase difference between cogging torque of inner and outer air gap of DRFPMG. Hence, the resultant cogging torque of DRFPMG is smaller than the cogging torque of inner or outer air gap.

The inner and outer effective air gap length of the DRFPMG are given by

(2) $$\begin{array}{c} g_{ei}=K_{Ci}g_{i},\\ g_{eo}=K_{Co}g_{o}, \end{array}$$

where *g*_*ei*_/*g*_*eo*_ is the effective inner/outer air gap length, *g*_*i*_/*g*_*o*_ is the inner/outer air gap length, and *K*_*Ci*_/*K*_*Co*_ is Carter's coefficient of inner/outer air gap.

Carter's coefficient of inner and outer air gap is

(3) KCi=τsiτsi−(2θi/Π){tan−1(θi/2gi)−(gi/θi)ln⁡[1+(θi/2gi)2]},KCo=τsoτso−(2θo/Π){tan−1(θo/2go)−(go/θo)ln⁡[1+(θo/2go)2]},

where *τ*_*si*_/*τ*_*so*_ is the slot pitch of inner/outer slots and *θ*_*i*_/*θ*_*o*_ is the slot opening width of inner/outer slots. The inner (*Ф*_*gi*_) and outer (*Ф*_*go*_) air gap flux of the DRFPMG are

(4) $$\begin{array}{c} \Phi_{gi}=B_{gi}L\tau_{pi},\\ \Phi_{go}=B_{go}L\tau_{po}, \end{array}$$

where *B*_*gi*_/*B*_*go*_ is the inner/outer air gap flux density, *L* is the axial length of machine, and *τ*_*pi*_/*τ*_*po*_ is the pole pitch of the inner/outer rotor.

The PM pole arc width plays vital role in the creation of cogging torque. The cogging torque is directly proportional to the square of the air gap flux. Thus, the change in PM pole arc width shifts away the maximum values of inner and outer cogging torque. The inner and outer air gap flux of the DRFPMG are given by (5) and (6), respectively:

(5) $$\begin{array}{c} \Phi_{gi}=\frac{\Pi }{2\sqrt{2}}\left(\frac{\beta_{i}+M_{\text{wi}}}{\beta_{i}}\right)\frac{B_{1gi}L\tau_{pi}}{\mathrm{Sin}\omega_{ei}} \end{array}$$

(6) $$\begin{array}{c} \Phi_{go}=\frac{\Pi }{2\sqrt{2}}\left(\frac{\beta_{o}+M_{wo}}{\beta_{o}}\right)\frac{B_{1go}L\tau_{po}}{\mathrm{Sin}\omega_{eo}}, \end{array}$$

where *β*_*i*_ is the inner rotor PM pole arc width, *β*_*o*_ is outer rotor PM pole arc width, *M*_*wi*_/*M*_*wo*_ is the arc width between two consecutive magnets of inner/outer rotor, *B*_1*gi*_/*B*_1*go*_ is the fundamental component of magnetic flux density of the inner/outer air gap, *τ*_*pi*_/*τ*_*po*_ is the pole pitch of inner/outer rotor, and *ω*_*ei*_/*ω*_*eo*_ is the electrical angular width of one-half of PM of the inner/outer rotor.

A section of FEA model of the DRFPMG is shown in Figure 2. In this model, the windings present in the stator were removed as they do not play an important role in the determination of cogging torque. The parameters *θ*_*i*_ and *θ*_*o*_ are the slot opening angular widths of inner stator slots and the outer stator slots, respectively.

Each rotor of the DRFPMG has its own identical PM poles equally spaced around it, respectively. The parameters *β*_*i*_ and *β*_*o*_ are the angular pole arc widths of the inner and the outer rotor PM, respectively. The arc length between two consecutive stator slot openings of the inner and outer stator slots is represented as *α*_*i*_ and *α*_*o*_, respectively.

**Figure 3:**
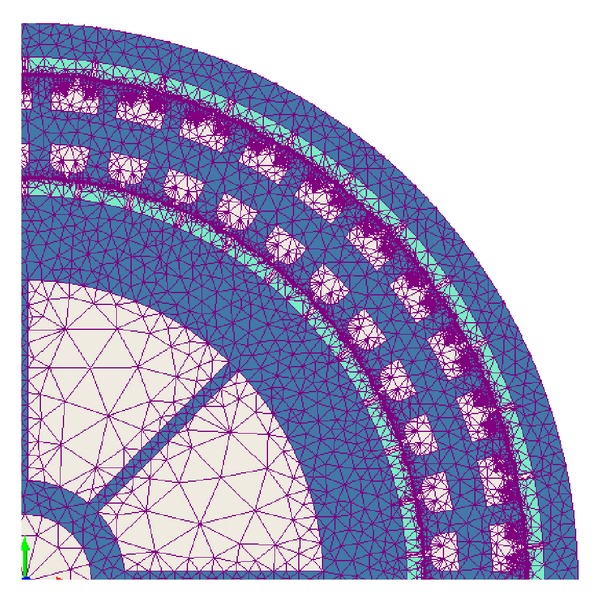
A section of DRFPMG FEM mesh.

A section of DRFPMG FEM mesh is shown in Figure 3. The mesh element size is two for outer rotor core, inner rotor core, and stator core. The mesh element size is one for inner rotor magnets and outer rotor magnets. The mesh element size is 0.5 for inner air gap and outer air gap. The computation domain totally consists of 112130 finite elements of triangle shape. The size of triangular elements was kept smaller in the air gap region as majority of the magnetic energy is found here.

**Figure 4:**
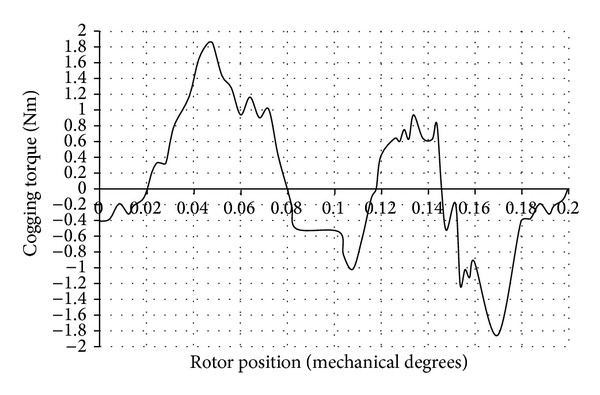
Cogging torque waveform of DRFPMG.

The cogging torque waveform obtained using two-dimensional (2D) magneto static FEA is shown in Figure 4. The CPU time taken for the computation of cogging torque was 14 hours and 24 minutes. The number of problems solved was 46. An Intel Pentium Dual Core CPU T4300 processor with 2.2 GHz frequency was used for the above computation.

The reluctance of the magnetic circuit varies as a function of the rotor angle. When the rotor was displaced from an initial, low reluctance position towards a higher reluctance, a torque resisting this displacement was experienced. When the rotor passed the point of the highest reluctance, the torque then tends to attract it to the next point of low reluctance and created a periodic torque waveform.

The peak value of the cogging torque present in the DRFPMG was observed to be 1.818 Nm. There were 46 periods of cogging torque in every 8 mechanical degrees of revolution of rotor. Thus, the periodicity of cogging torque waveform was 0.173 degrees for the DRFPMG considered.

## 4. Reduction of Cogging Torque in DRFPMG

The FEA of cogging torque reduction by changing slot opening width, changing magnet pole arc width, shifting of slot openings, and combination of these methods are discussed here. The cogging torque values obtained through all these methods are given in Tables 1 and 2.

### 4.1. Changing Slot Opening Width

**Figure 5:**
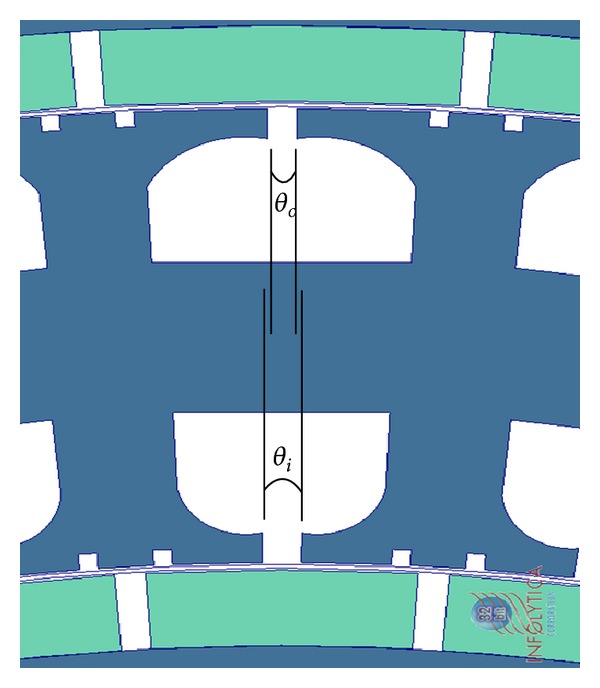
Section of FEA model showing change in slot opening width.

The equal angular widths of the slot opening of the inner periphery (*θ*_*i*_) and outer periphery (*θ*_*o*_) of the stator gave a large slot opening for the slots on the outer periphery of the stator. Due to this, the resultant cogging torques of the inner and outer air gaps were in phase with each other. The total cogging torque produced was much higher. In this method, the angular width of the slots of outer periphery of the stator was reduced as shown in Figure 5. Hence, the slot opening width of inner slots (*b*_*i*_) is 1.5 mm and the slot opening width of outer slots (*b*_*o*_) is 1.35 mm.

**Figure 6:**
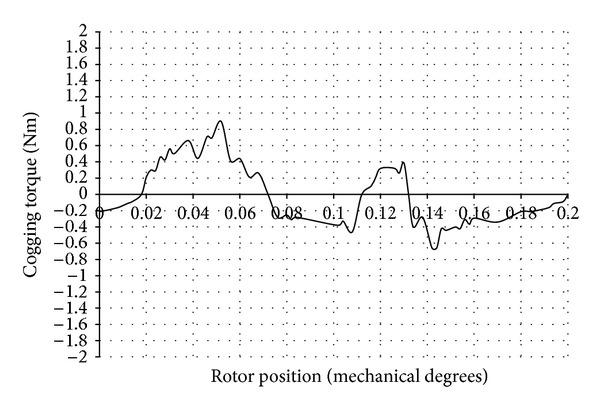
Cogging torque waveform after changing slot opening width.

Now the cogging torque waveforms of inner and outer air gaps were different. Hence, the total cogging torque got reduced. The values of cogging torque obtained before and after changing the slot opening width are given in Table 1. The cogging torque waveform after changing the slot opening width obtained through FEA is shown in Figure 6. The percentage reduction of cogging torque is 51.1.

### 4.2. Changing Magnet Pole Arc Width

**Figure 7:**
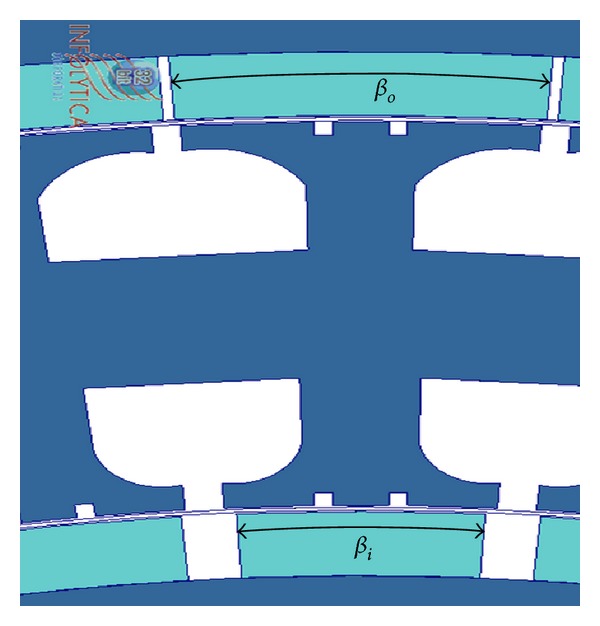
Section of FEA model showing change in magnet pole arc width.

In conventional PM generators, the cogging torque is reduced if the permanent magnet width is almost equal to an integer number of slot pitches. But, in DRFPMG, the cogging torque was reduced by keeping different angular arc widths for the inner and outer rotor magnets. The angular pole arc width of inner rotor magnets (*β*_*i*_) was made smaller than the angular pole arc width of outer rotor magnets (*β*_*o*_) as shown in Figure 7. Due to this, the pole arc width of inner rotor magnets (*l*_*i*_) is 8.99 mm and the pole arc width of outer rotor magnets (*l*_*o*_) is 20.32 mm.

**Figure 8:**
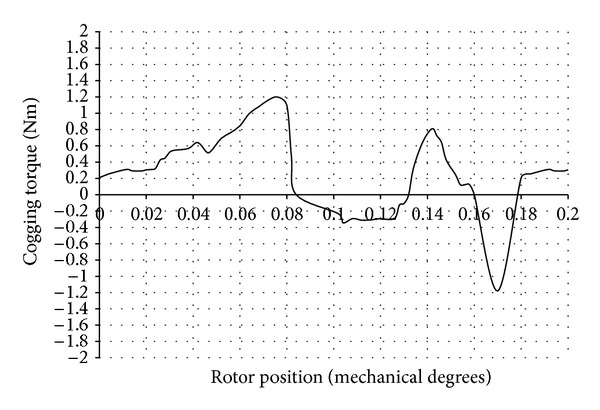
Cogging torque waveform after changing magnet pole arc width.

The cogging torque values obtained before and after changing the magnet pole arc width are given in Table 1. The cogging torque waveform after changing the magnet pole arc width obtained through FEA is shown in Figure 8. The percentage reduction of cogging torque by this method is 33.99.

### 4.3. Shifting of Slot Openings

**Figure 9:**
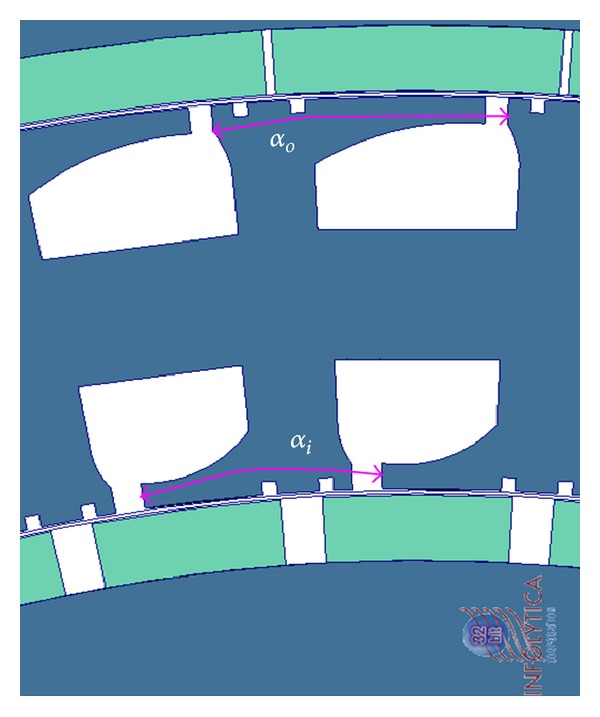
Section of FEA model showing shifted slot opening.

In DRFPMG, if the slots were aligned in radial direction, the cogging torques produced by both of the air gaps were in phase with each other. Hence, the net cogging torque was more than each individual component. In this method, the arc distance between two consecutive inner slot openings (*α*_*i*_) and the arc distance between two consecutive outer slot openings (*α*_*o*_) were shifted by half of the slot pitch. Thus, the inner and outer slot openings were shifted away from each other by half slot pitch each as shown in Figure 9. Now some portions of the cogging torque produced by the outer slots were cancelled by the cogging torque produced by inner slots.

**Figure 10:**
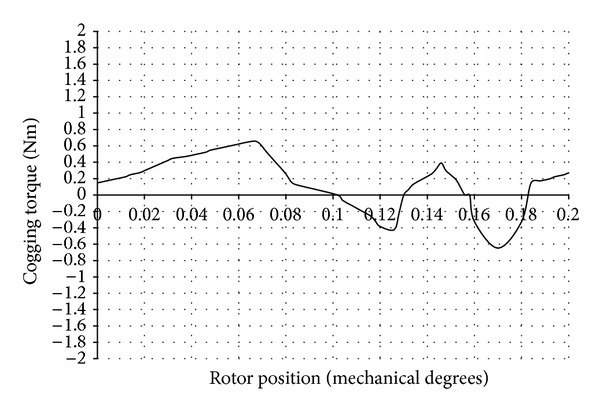
Cogging torque waveform after shifting of slot openings.

The cogging torque values obtained before and after shifting the slot openings are given in Table 1. The cogging torque waveform after shifting the slot openings, obtained through FEA, is shown in Figure 10. The percentage reduction of cogging torque is 64.3.

### 4.4. Changing Magnet Pole Arc Width and Slot Opening Width and Shifting of Slot Openings

**Figure 11:**
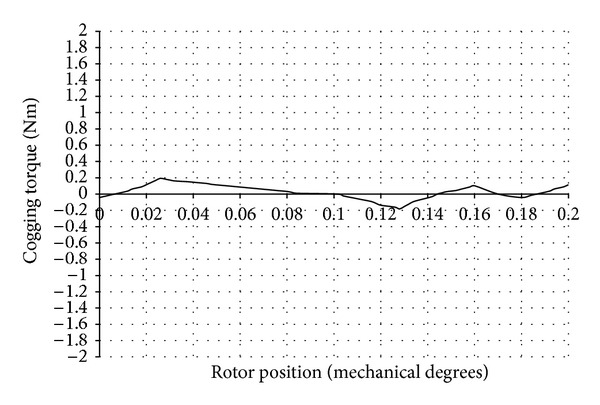
Cogging torque waveform after combined effect.

A combination of three methods, changing magnet pole arc width, changing slot opening width, and shifting of slot openings, was applied simultaneously (combined effect) for further reduction of cogging torque. The cogging torque values obtained before and after combined effect are given in Table 2. The cogging torque waveform after combined effect, obtained through FEA, is shown in Figure 11.

The percentage reduction of cogging torque by this method is 89.33 which is greater than that achieved by all the above methods.

## 5. Induced emf of DRFPMG

**Figure 12:**
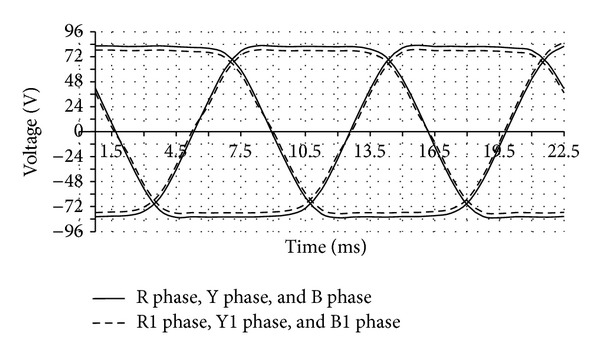
Induced emf before and after cogging torque reduction.

After the application of each method of cogging torque reduction in DRFPMG, the change in induced emf has been evaluated. A transient 2D with motion FEA simulation has been carried out using MagNet Software to determine the no load induced emf. Before the application of cogging torque reduction methods, the emf induced in DRFPMG was 78.4 V. After the change in slot opening width, the transient 2D with motion was run. The induced emf observed was 77.24 V. Similarly, the induced emf observed for changing magnet pole arc width and shifting of slot openings and combined effect were 76.55 V, 77.55 V, and 76.14 V, respectively. The no load induced emf obtained before and after cogging torque reduction by combined effect is shown in Figure 12. The phases RYB were obtained before cogging torque reduction and R1Y1B1 phases were obtained after cogging torque reduction. The percentage reduction of induced emf and cogging torque in each method is given in Table 3. It was found that up to 2.88 percent of the induced emf got reduced due to the geometrical alterations made while reducing the cogging torque.

## 6. Fabrication of DRFPMG

**Figure 13:**
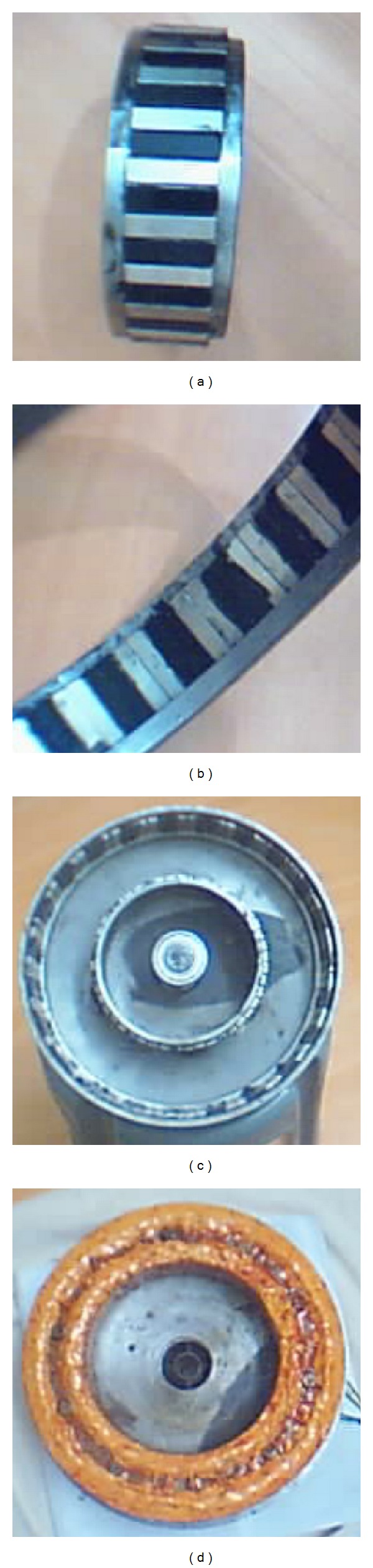
Rotors and stator of DRPMG prototype.

A prototype of 1 kW, 120 rpm DRFPMG was fabricated. The important specifications of the prototype are given in Table 4. The rotor cores and stator core were made up of silicon steel laminations having 0.45 mm thickness. The size of NdFeB permanent magnets used in inner and outer rotor was 40 × 10 × 5 mm and 40 × 20 × 5 mm, respectively.  Figure 13(a) shows the inner rotor with surface mounted magnets on its outer periphery.  Figure 13(b) shows the section of outer rotor with surface mounted magnets on its inner periphery.  Figure 13(c) shows the dual rotor assembly.  Figure 13(d) shows the wound stator. The number of coils per phase was 15 each in both inner and outer periphery of stator. In the shifting of slot openings method, the cogging torque was suitably reduced and the induced emf is better than other methods as seen in Table 3. Hence, the shifting of slot openings method was adopted in the experimental prototype. The slot openings of the stator were shifted in order to reduce the cogging torque. The arc distance between two consecutive inner and outer slot openings was shifted by 6.01 mm anticlockwise and 10.11 mm clockwise, respectively.

## 7. Experimental Results and Discussion

**Figure 14:**
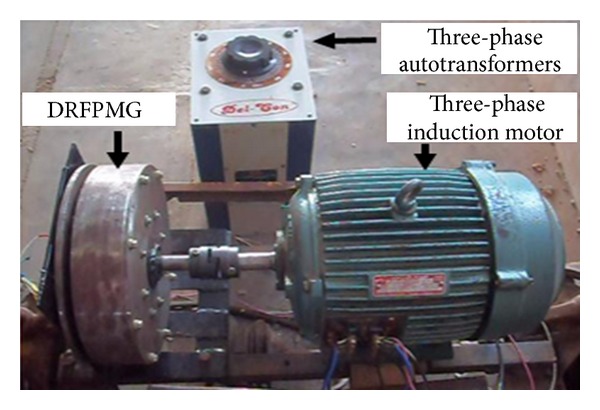
Experimental setup.

The experimental measurement of induced emf and power and cogging torque of DRFPMG prototype was carried out using a laboratory set-up shown in Figure 14.

The set-up consisted of a 5 HP three-phase induction motor (IM) mechanically coupled with the 1 kW DRFPMG prototype. The three-phase IM was operated as a prime mover. A three-phase autotransformer was used to control the speed of the three-phase IM. The entire set-up was used as a laboratory model of direct coupled standalone wind energy system employing a dual rotor radial flux permanent magnet generator, for power generation.

**Figure 15:**
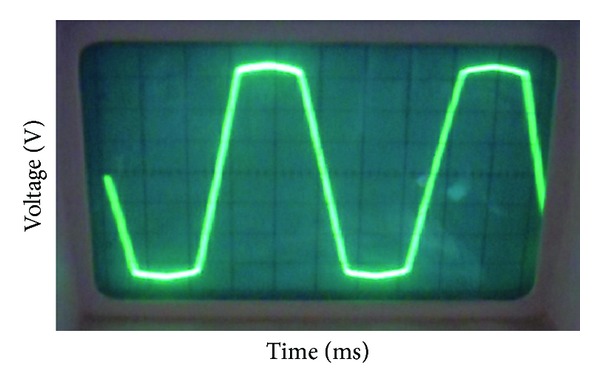
No load induced EMF.

The prototype of DRFPMG was run at a speed of 120 rpm. As the dual rotor rotates, the flux produced by the inner rotor magnets was cut by three-phase windings of the inner slots of the stator core. Simultaneously, the flux produced by the outer rotor magnets was cut by three-phase windings of the outer slots of the stator core. As the three-phase windings of inner and outer slots of stator are in series connection, the induced emf were added up. The no load induced emf waveform of DRFPMG prototype is shown in Figure 15. At 120 rpm, the peak voltage measured was 78.4 V and frequency was 46 Hz. The power capacity of the DRFPMG prototype was tested using lamp loads. At 120 rpm, under full load condition, the output power of DRFPMG prototype was 1044 W.

The cogging torque of DRFPMG prototype was measured with piezo electric reaction torque sensor. The sensor was mounted beneath the housing of the motor. To measure the cogging torque, the rotor of DRFPMG prototype was turned mechanically with the help of the three-phase induction motor. The cogging torque was observed to be 0.591 Nm. The FEA and experimental results of no load induced emf, power capacity, and cogging torque are compared in Table 5. The values are in good agreement.

## 8. Conclusion

The important methods for reducing the cogging torque concerned with dual rotor permanent magnet generator have been investigated. The 2D static and transient with motion FEA simulations were carried out. In FEA, the percentage reduction in cogging torque by changing slot opening width, changing magnet pole arc width, shifting of slot openings, and combined effect was 51.1, 33.99, 64.3, and 89.33, respectively. The corresponding percentage reductions in induced emf were 1.48, 2.36, 1.08, and 2.88, respectively. The reduction of cogging torque was carried out without major change in the induced emf of the generator. A prototype of 1 kW, 120 rpm DRFPMG was fabricated. The shifting of slot opening method was implemented in the fabrication to reduce the cogging torque. The FEA and experimental results were tabulated. The experimental results have shown good agreement with the simulation predictions.

## Figures and Tables

**Table 1:** Cogging torque values obtained before and after applying the different cogging torque reduction methods individually.

S. number	Cogging torque reduction method	Before applying cogging torque reduction method			After applying cogging torque reduction method		
1	Changing slot opening width	*b*_*i*_ (mm)	*b*_*o*_ (mm)	*T*_cog_ (Nm)	*b*_*i*_ (mm)	*b*_*o*_ (mm)	T_cog_ (Nm)
		1.5	2.5	1.818	1.5	1.35	0.889
2	Changing magnet pole arc width	*l*_*i*_ (mm)	*l*_*o*_ (mm)	*T*_cog_ (Nm)	*l*_*i*_ (mm)	*l*_*o*_ (mm)	T_cog_ (Nm)
		10.68	20.32	1.818	8.99	20.32	1.2
3	Shifting of slot openings	*α*_*i*_	*α*_*o*_	T_cog_ (Nm)	*α*_*i*_	*α*_*o*_	T_cog_ (Nm)
		Not shifted		1.818	Shifted anticlockwise by 6.01 mm	Shifted clockwise by 10.11 mm	0.612

**Table 2:** Cogging torque values obtained before and after combined effect.

Parameters	Before reduction	After applying combined effect
*b*_*i*_ (mm)	1.5	1.5
*b*_*o*_ (mm)	2.5	1.35
*l*_*i*_ (mm)	10.68	8.99
*l*_*o*_ (mm)	20.32	20.32
*α*_*i*_	Not shifted	Shifted anticlockwise by 6.01 mm
*α*_*o*_	Not shifted	Shifted clockwise by 10.11 mm
T_cog_ (Nm)	1.818	0.194

**Table 3:** Percentage reduction in cogging torque and induced emf.

S. number	Cogging torque reduction method	Cogging torque reduction (%)	Induced emf reduction (%)
1	Changing slot opening width	51.10	1.48
2	Changing magnet pole arc width	33.99	2.36
3	Shifting of slot openings	64.30	1.08
4	Combined effect	89.33	2.88

**Table 4:** Important specifications of DRFPMG prototype.

Symbol	Values	Unit
*P*_*R*_	1	kW
*N*	120	rpm
*f*	46	Hz
*V*	78	V
*D*_*roo*_	0.3115	m
*D*_*roi*_	0.3015	m
*D*_*rii*_	0.1510	m
*D*_*rio*_	0.1710	m
*D*_*si*_	0.1722	m
*D*_*so*_	0.2897	m
*L*	0.04	m
*T*_*m*_	0.005	m
*N*_*si*_	45	—
*N*_*so*_	45	—
*N*_*pri*_	46	—
*N*_*pro*_	46	—

*P*_*R*_ is the rated power, *N* is rated speed, *f* is frequency, *V* is output voltage at no load, *D*_*roo*_ is outer diameter of outer rotor, *D*_*roi*_ is inner diameter of outer rotor, *D*_*rii*_ is inner diameter of inner rotor, *D*_*rio*_ is outer diameter of inner rotor, *D*_*si*_ is stator inner diameter, *D*_*so*_ is stator outer diameter, *L* is effective axial length, *T*_*m*_ is thickness of magnet, *N*_*si*_ is number of inner stator slots, *N*_*so*_ is number of outer stator slots, *N*_*pri*_ is number of inner rotor poles, and *N*_*pro*_ is number of outer rotor poles.

**Table 5:** FEA and experimental results.

Parameter	FEA	Experiment	Unit
*V*	78.8	78.4	Volts
*T*_cog_	0.612	0.591	Nm
*P*_*R*_	1.032	1.044	kW
